# Exploring plant diversity through soil DNA in Thai national parks for influencing land reform and agriculture planning

**DOI:** 10.7717/peerj.11753

**Published:** 2021-08-02

**Authors:** Maslin Osathanunkul, Nipitpong Sawongta, Wittaya Pheera, Nikolaos Pechlivanis, Fotis Psomopoulos, Panagiotis Madesis

**Affiliations:** 1 Research Center in Bioresources for Agriculture, Industry and Medicine, Chiang Mai University, Chiang Mai, Thailand; 2 Faculty of Science, Department of Biology, Chiang Mai University, Chiang Mai, Thailand; 3 Institute of Applied Biosciences (INAB), Centre for Research & Technology Hellas (CERTH), Thessaloniki, Greece; 4 Development and Molecular Biology, School of Biology, Department of Genetics, Aristotle University of Thessaloniki, Thessaloniki, Greece; 5 Crop Production and Rural Environment, Laboratory of Molecular Biology of Plants, Department of Agriculture, University of Thessaly, Volos, Magnesia, Greece

**Keywords:** Agricultural expansion, Environmental DNA, Metabarcoding, Plant diversity, National Park

## Abstract

**Background:**

The severe deforestation, as indicated in national forest data, is a recurring problem in many areas of Northern Thailand, including Doi Suthep-Pui National Park. Agricultural expansion in these areas, is one of the major drivers of deforestation, having adverse consequences on local plant biodiversity. Conserving biodiversity is mainly dependent on the biological monitoring of species distribution and population sizes. However, the existing conventional approaches for monitoring biodiversity are rather limited.

**Methods:**

Here, we explored soil DNA at four forest types in Doi Suthep-Pui National Park in Northern Thailand. Three soil samples, composed of different soil cores mixed together, per sampling location were collected. Soil biodiversity was investigated through eDNA metabarcoding analysis using primers targeting the P6 loop of the plastid DNA *trnL* (UAA) intron.

**Results:**

The distribution of taxa for each sample was found to be similar between replicates. A strong congruence between the conventional morphology- and eDNA-based data of plant diversity in the studied areas was observed. All species recorded by conventional survey with DNA data deposited in the GenBank were detected through the eDNA analysis. Moreover, traces of crops, such as lettuce, maize, wheat and soybean, which were not expected and were not visually detected in the forest area, were identified. It is noteworthy that neighboring land and areas in the studied National Park were once used for crop cultivation, and even to date there is still agricultural land within a 5–10 km radius from the forest sites where the soil samples were collected. The presence of cultivated area near the forest may suggest that we are now facing agricultural intensification leading to deforestation. Land reform for agriculture usage necessitates coordinated planning in order to preserve the forest area. In that context, the eDNA-based data would be useful for influencing policies and management towards this goal.

## Introduction

Thailand has been ambitiously aiming to have a forest coverage of 40% throughout the country, but this goal, set in 1985, has yet to be accomplished. However, satellite data to assess forest changes in tree density indicate that areas surrounding forests, e.g., agricultural areas and neighboring settlements, had a net gain in tree density (Reytar, Stolle & Anderson, 2019). Forest loss is nevertheless outpacing forest gain in Thailand. Forest coverage has declined from approximately 70% in 1950 to 31% in 2018 (Royal Forest Department, 1999). Deforestation has been occurring to an extreme degree in many parts of Northern Thailand (Delang, 2002; Virapongse, 2017).

Numerous studies have shown that deforestation results in a loss of plant diversity (Barlow et al., 2016; Giam, 2017; Decaëns et al., 2018). Agricultural expansion is one of the major drivers for deforestation in Thailand and several other countries in Asia, which resulted in plant biodiversity loss having adverse effects on ecology and climate (Jayathilake et al., 2021; Hosonuma et al., 2012). Thailand was endowed with cultivable land covering over 50% of the country’s land area (Land Development Department, 2012). As a result, the change from subsistence agriculture to cash crops cultivation, required an expansion of the agricultural land base (Jamroenprucksa, 2007). Over the past few decades, the area of agricultural land has expanded at the expense of the forest biodiversity. Conservation of biodiversity depends on the biological monitoring of plant species distribution not only for preserving threatened species, but also for maintaining the resilience and health of the ecosystems. Species monitoring in empirical ecological studies has thus far been relied on the morphology-based species identification. This is usually performed by morphological investigation and by counting individuals in the field (Elliott & Jonathan Davies, 2014). Obtaining biodiversity data solely by existing conventional approaches is hindered by several factors, including lack of diagnostic characters, the requirement of taxonomic expertise, as well as the associated expenses (Yoccoz et al., 2012; Fahner et al., 2016). In order to improve our understanding on the state of forest diversity, novel assessment methods should be applied characterized by sensitivity, effectiveness and reliability.

The latest sequencing technologies have paved the way in diversity studies. High-throughput sequencing (HTS) of DNA barcode amplicons (DNA metabarcoding) has proved to be a robust, accurate and efficient approach to survey biodiversity (Epp et al., 2012; Taberlet et al., 2012a; Yoccoz et al., 2012). Traces of living organisms including animals, plants and fungi can be accumulated and detected in the environmental DNA (eDNA). eDNA metabarcoding has been used to analyze the complex DNA from environmental samples like soil, water, air and feces (Drummond et al., 2015; Creer et al., 2016). Several markers are now available for use in the eDNA metabarcoding for various groups of organisms (Drummond et al., 2015; Fahner et al., 2016). Given that eDNA metabarcoding is a powerful technique offering many possibilities, we have used this approach to explore soil eDNA, which is expected to reflect taxonomic richness and diversity of plants in the studied area.

Deforestation is an ongoing problem in Northern Thailand and especially the Doi Suthep-Pui National Park (Marod et al., 2014). Doi Suthep-Pui National Park is the 24th national park, located in Chiang Mai Province. There are different altitudes in the National Park ranging from 330 m to 1,685 m ASL, resulting to a great variety of flora and fauna. The forest ecosystem of the Doi Suthep-Pui National Park is known for its wide indigenous genetic diversity. The major forest types represented in Doi Suthep-Pui National Park include the deciduous forest of the lowlands (deciduous dipterocarp, bamboo deciduous forest and mixed evergreen deciduous forest) and the evergreen forest of the uplands (Maxwell & Elliott, 2001). Although the national park is protected by various conservation measures, it is still facing plant diversity loss due to deforestation. Thus, eDNA data on species diversity is required for effective monitoring of biodiversity in this area for conservation and management purposes.

## Materials & Methods

### Soil sampling

Soil samples were collected from four forest types in Doi Suthep-Pui National Park in Northern Thailand. The locations where the soil samples were taken are indicated in Table 1. We collected three samples (each composed of many soil cores mixed together) per sampling location, which were subsequently used for the DNA extraction. Field permits were granted by the Department of National Parks, Wildlife and Plant Conservation (Field Permit number: 0907.4/2529).

**Table 1:** Locations were the soil samples were collected.

**Abbreviation**	**Communities**	**Latitude**	**Longitude**	**Altitude**
CA	coniferous	18°49′51”N	98°53′33”E	1,342
HE	hill evergreen	18°49′36”N	98°53′55”E	1,214
DD	dry dipterocarp	18°47′15”N	98°55′10”E	827
DE	dry evergreen	18°47′16”N	98°55′11”E	809

### DNA extraction and sequence analyses

Extracellular DNA was extracted twice from 15 g of soil per soil core as described previously (Taberlet et al., 2012b). Air-dried soil sample was mixed with 15 ml of saturated phosphate buffer (Na_2_HPO_4_; 0.12 M, pH ≈ 8) for 15 min followed by centrifugation for 10 min at 10,000 g. Approximately 400 µl of the supernatant were used as starting material for extraction by the NucleoSpin Soil kit (MACHEREY–NAGEL, Germany), following the manufacturer’s instructions omitting the cell lysis step.

Soil biodiversity was investigated through eDNA metabarcoding analysis using primers targeting the P6 loop of the plastid DNA *trnL* (UAA) intron using the g (5′-GGGCAATCCTGAGCCAA-3′) and h (5′-CCATTGAGTC TCTGCACCTATC-3′) primers (Yoccoz et al., 2012), which were 5′- labelled for each soil core sample with a unique eight-nucleotide tag. PCR negative controls were included for a quality check of the amplifications. The sequencing was conducted by 2  × 125 base pairs, pair-end sequencing. The sequences of DNA were filtered with OBITools software (Boyer et al., 2016) following the procedure detailed in Pansu et al. (2015). The initial step of the process was to join the forward and reverse reads, using the OBITools suite. The Illumina paired-end tool of the suite aligned the two reads of the sequenced pair-end library, returning either the consensus sequence, or when there was no overlap, the concatenation of the forward and reverse-complement sequence reads.

The next step was to distinguish between sequences from different PCR products (from 24 extractions) pooled in the same sequencing library. The Next-Generation Sequencing filter tool from the suite was used in this instance, taking as input the aligned/merged reads and a file containing tag pairs information corresponding to each sample. The results consisted of sequence records with their sequence trimmed of the primers and tags and annotated with the corresponding experiment and sample. Sequences for which the tags and primers have not been well identified, and which are thus unassigned to any sample, are stored in a different file and tagged as erroneous sequences by the tool.

The first step of the downstream analysis was to de-multiplex the single FASTQ file into multiple sample-specific FASTQ-formatted files. This was performed using standard Linux commands for splitting the original file. After demultiplexing, quality trimming was performed with Trim Galore tool using the default parameters (default Phred score 20). The trimmed samples were imported into QIIME 2 (version 2020.8) as single-end files. All samples were denoised, dereplicated and filtered for chimeras with the DADA2 plugin. No singletons are reported as DADA2 does not call singletons. In Table 2 the total number of processed reads during the DADA2 step is presented. For each sample between 80% to 98% of the raw reads were passed as non-chimeric. The resulted reads were clustered with the de novo clustering method having percent identity of 99%. The final step was to annotate the final OTUs with the taxonomy based on homology with sequences in the National Center for Biotechnology Information (NCBI) non-redundant (NR) database. The NR database is compiled by the NCBI as a protein database for BLAST searches. It contains non-identical sequences from GenBank CDS translations, PDB, Swiss-Prot, PIR, and PRF. The strengths of NR are that it is comprehensive and frequently updated. This annotation step was performed using the HERMES tool (Kintsakis et al., 2017) to run a full BLAST comparison with NR, and keeping the single best hit per sequence. The GenInfo Identifier (GI) number, a simple series of digits that are assigned consecutively to each sequence record processed by NCBI, of each hit was consequently connected to the corresponding taxonomy and the query sequence (i.e., original OTU) was annotated with the produced taxonomy.

**Table 2:** Number of processed reads over the downstream analysis.

**Sample-id**	**Input (after quality trimming)**	**Filtered**	**Input passed filter (%)**	**Denoised**	**Non-chimeric**	**Input non-chimeric (%)**
CA1	186,403	185,030	99.26	184,613	168,120	90.19
CA2	114,641	112,908	98.49	111,947	102,832	89.7
CA3	264,423	256,404	96.97	255,978	232,368	87.88
DD1	188,652	186,650	98.94	185,492	177,079	93.87
DD2	173,521	173,106	99.76	172,795	170,795	98.43
DD3	174,168	173,531	99.63	173,459	146,102	83.89
DE2	143,186	142,606	99.59	142,354	139,775	97.62
DE3	175,162	174,653	99.71	174,230	172,227	98.32
HE1	163,084	159,777	97.97	158,689	140,204	85.97
HE2	198,672	192,739	97.01	191,885	180,297	90.75
HE3	132,638	129,097	97.33	128,393	121,062	91.27

Within-community (alpha) diversity was estimated for every sample, using the *phyloseq* package in R (McMurdie & Holmes, 2013). As an input to the function the clustered OTU table was used in which singletons were excluded. For this reason, the Shannon index was used in order to avoid estimates that are highly dependent on the number of singletons.

## Results

**Figure 1:**
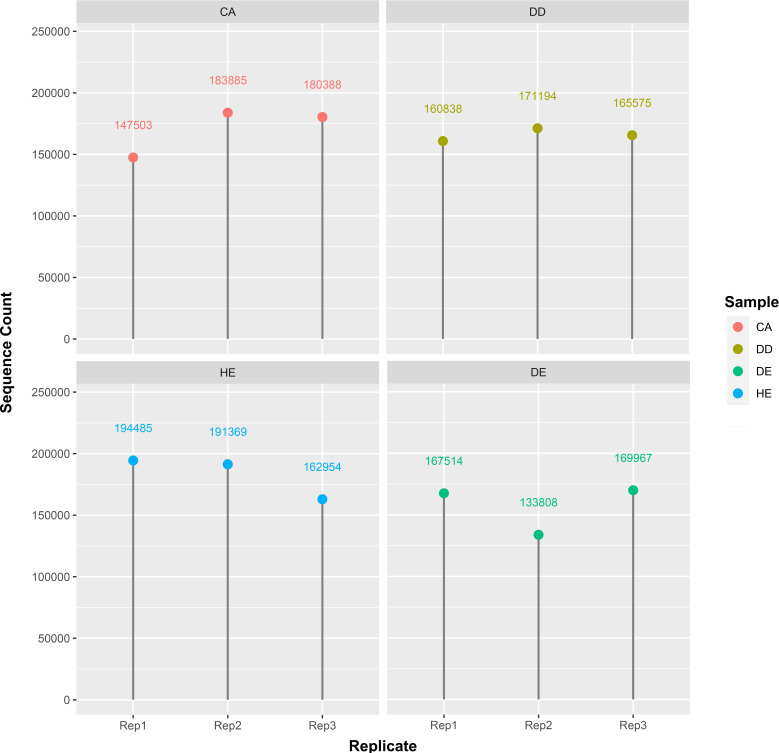
Distribution of the sequence count per sample. There are four samples which are CA: coniferous, DD: dry dipterocarp, DE: dry evergreen, and HE: hill evergreen with three replicates per sample.

In this study, we used eDNA metabarcoding method to explore plant diversity from soil sampled at four forest types in Doi Suthep-Pui National Park. We obtained a total of 2,031,271 g/h sequence reads from 12 different samples including Coniferous (CA1-3), Dry dipterocarp (DD1-3), Dry evergreen (DE1-3), Hill evergreen (HE1-3) soils (Fig. 1). Numbers of processed reads over the downstream analysis of each sample are shown in Table 2.

**Figure 2:**
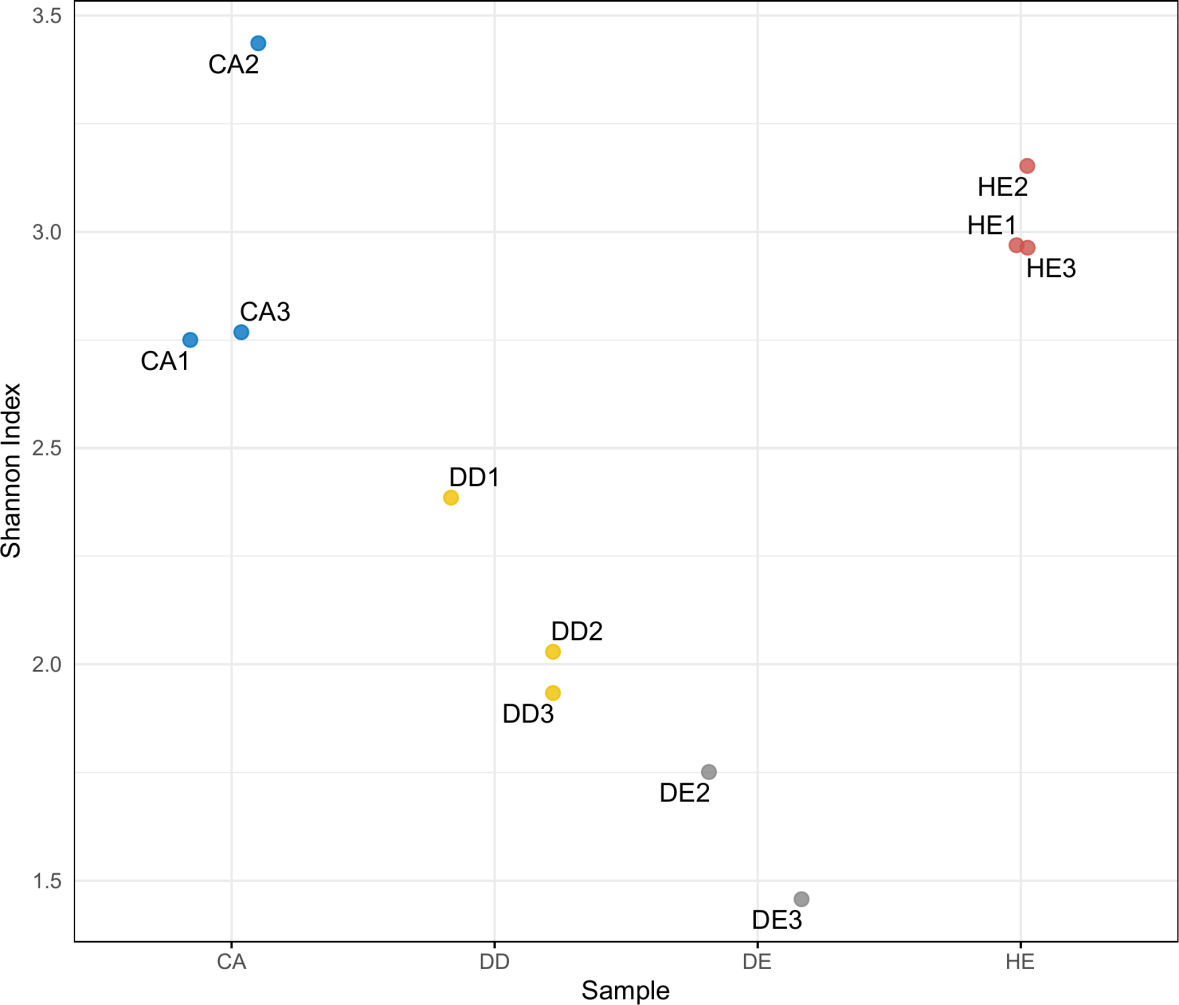
Within-community (alpha) diversity measured with the Shannon index as a function of the sample group (CA, DD, DE and HE).

**Figure 3:**
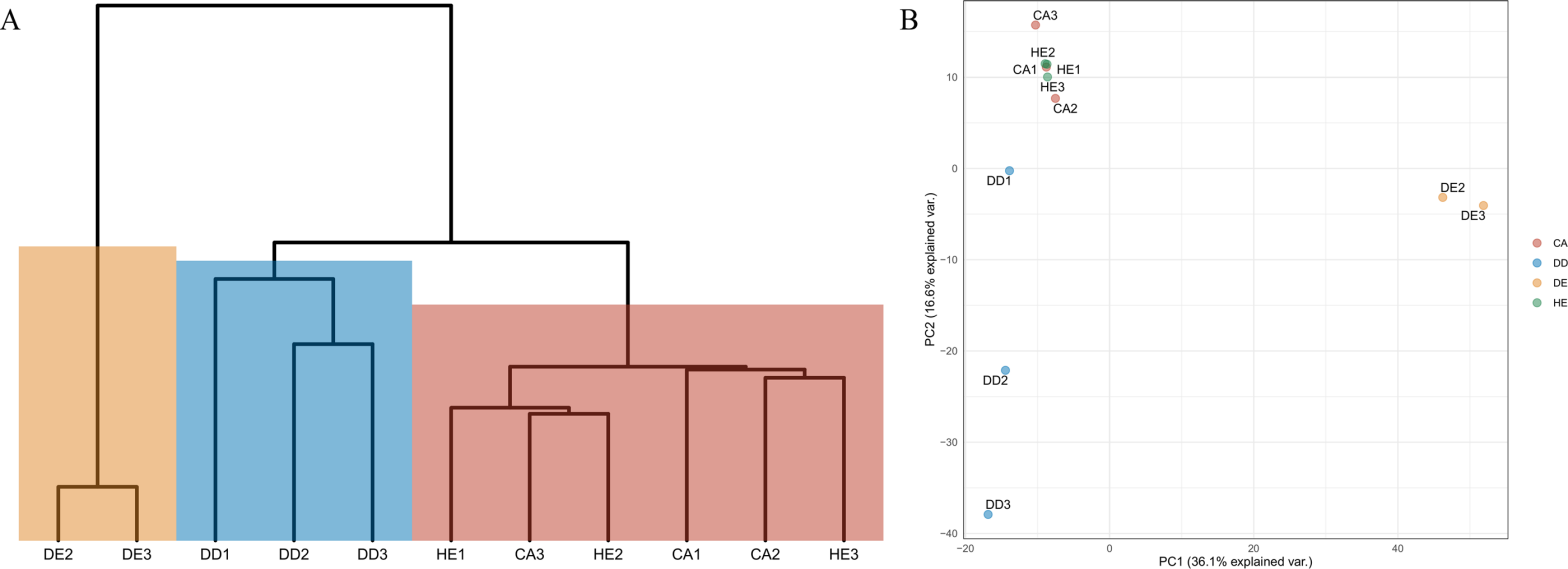
(A) Hierarchical clustering of the samples based on the normalized (Total Sum Scaling normalization–TSS) OTU table. The Euclidean distance metric and the ward. D clustering method have been used. (B) The PC1–PC2 two-dimensional space along with the corresponding explained variance.

Differences in diversity between samples were measured using the Shannon diversity index (Fig. 2). The alpha diversity results indicate that Coniferous (CA) and Hill Evergreen (HE) samples present higher mean species diversity, in comparison to Dry Dipterocarp (DD) and Dry Evergreen (DE) samples. Moreover, we performed Principal Component Analysis and Hierarchical clustering in order to investigate how are the samples clustered. To this end the OTU table was used on which Total Sum Scaling (TSS) normalization was applied. In Fig. 3A. The result of Hierarchical clustering with the Euclidean distance method and the Ward.D clustering method is shown. In Fig. 3B. all samples are projected in PC1–PC2 dimensional space. In both cases the Coniferous (CA) and Hill Evergreen (HE) samples are grouped together, in agreement with the alpha diversity results.

According to the species annotation results (1. 7578  × 10^6^ reads), the statistics of sequence number were placed in different classification levels i.e., genus and species. The dominant taxa among all samples, which were obtained from conventional survey and our analyses are displayed in Table 3. Interestingly, there are 31 plants that could not be identified at the species level and nine that were only visually identified but could not be detected by the eDNA method (Table 3). Analysis of DNA data from these 40 species in the GenBank database showed that none of those species had the *trnL* sequence deposited (Supplementary data 1).

**Table 3:** Plant species found by traditional survey and soil eDNA metabarcoding.

**Family**	**Species**	**Conventional survey**				**Soil eDNA**
		**CA**	**DD**	**DE**	**HE**	
Actinidiaceae	*Saurauia napaulensis* DC.	–	–	–	x	x
	*Saurauia roxburghii* Wall.	x	x	–	x	x
Anacardiaceae	*Spondias pinnata* (L.f.) Kurz	–	–	x	–	x
Annonaceae	*Melodorum fruticosum* Lour	–	–	x	–	x
	*Miliusa lineata* Alston	–	–	x	–	x
Bignoniaceae	*Oroxylum indicum* vent.	–	–	x	–	x
	*Stereospermum neuranthum* Kurz	x	–	–	x	xx
Burseraceae	*Protium serratum* Engler	x	–	x	x	xx
Compositae	*Vernonia volkameriaefolia* Wall. ex DC.	x	–	–	x	xx
Dipterocarpaceae	*Dipterocarpus alatus* Roxb. ex G.Don	–	–	x	–	x
	*Dipterocarpus obtusifolius* Teijsm. ex Miq.	x	x	x	–	x
	*Dipterocarpus tuberculatu* s Roxb.	–	x	–	–	x
	*Dipterocarpus turbinatus* Gaertn. f.	x	x	x	–	x
	*Hopea odorata* Roxb.	–	x	x	–	x
	*Shorea obtusa* Wall. ex Blume	–	x	–	x	xx
	*Shorea roxburghii* G. Don	–	x	x	x	x
	*Shorea siamensis* Miq.	–	x	–	–	x
Ebenaceae	*Diospyros ehretioides* Wall. ex G. Don	–	x	–	–	x
Elaeocarpaceae	*Elaeocarpus sphaericus* Schum	–	–	–	x	x
	*Elaeocarpus stipularis* Bl.	x	–	x	x	x
Ericaceae	*Vaccinium sprengelii* Sleumer	x	x	–	x	xx
Euphorbiaceae	*Antidesma bunius* (L.) Spreng. var. bunius	x	–	–	x	–
	*Antidesma sootepense* Craib	–	x	–	x	–
	*Baccaurea ramiflora* Lour.	–	–	x	x	x
	*Balakata baccata* (Roxb.) Esser	–	x	–	–	x
	*Bischofia javensis* Blume	–	–	–	x	–
	*Bridelia affinis* Craib	–	x	–	–	x
	*Bridelia glauca* Blume	x	–	–	x	x
	*Bridelia retusa* (L.) A.Juss	–	x	–	x	x
	*Mallotus barbatus* Muell. Arg	x	–	–	x	x
	*Mallotus paniculatus* Muell. Arg	–	–	x	x	x
Fagaceae	*Castanopsis acuminatissima* Rehd.	x	x	x	x	xx
	*Castanopsis calathiformis* Kurz	x	–	x	x	x
	*Castanopsis diversifolia* King	x	x	x	x	xx
	*Castanopsis purpurea* Barnett	x	x	–	–	xx
	*Lithocarpus elegans* Hatus. ex Soepadmo	x	x	x	x	xx
	*Lithocarpus fenestratus* Rehd	–	–	x	x	x
	*Lithocarpus finetii* A. Camus	x	–	–	x	xx
	*Lithocarpus lindleyanus* A. Camus	x	x	–	x	xx
	*Lithocarpus tenuinervis* A. Camus	x	x	–	x	xx
	*Lithocarpus thomsonii* Rehd.	x	–	x	x	xx
	*Quercus brandisiana* Kurz	–	x	–	x	x
	*Quercus helferiana* A. DC.	x	–	x	x	xx
	*Quercus kerrii* Craib	x	x	x	x	xx
	*Quercus kingiana* Craib	x	x	x	x	xx
Flagellariaceae	*Flagellaria indica* Linn.	–	–	x	–	x
Gnetaceae	*Gnetum montanum* Markgr.	x	–	x	x	x
Hypericaceae	*Mesua ferrea* Linn.	–	–	x	–	x
Irvingiaceae	*Irvingia malayana* Oliv. ex A.W.Benn.	–	–	x	–	xx
Juglandaceae	Engelhardtia serrata Blume	x	–	x	x	x
	Engelhardtia spicata Blume var. colebrookeana (Lindl. ex. Wall.) Kuntze	x	–	–	x	x
Labiatae	*Clerodendrum serratum* (L.) Moon var. wallichii C.B. Clarke	x	x	x	x	x
	*Clerodendrum viscosum* Vent.	–	–	x	–	x
Lauraceae	*Actinodapne henryi* Gamble	–	–	–	x	–
	*Cryptocarya pallens* Kosterm	x	–	–	x	xx
	*Lindera metcalfiana* Allen	x	–	x	x	xx
Leguminosae	*Acacia megaladena* Desv. var. megaladena	–	–	x	x	xx
	*Dalbergia cana* Graham ex Kurz	–	x	–	x	x
	*Dalbergia cultrata* Graham ex Benth.	x	x	x	x	x
	*Dalbergia oliveri* Gamble	–	x	–	–	x
	*Dalbergia ovata* Grah.	x	–	–	x	xx
	*Desnodium megaphullum* Zoll	x	x	–	x	–
	*Indigofera caloneura* Kurz.	x	–	–	–	xx
	*Millettia pachycarpa* Benth.	–	–	–	x	x
	*Pterocarpus macrocarpus* Kurz	–	x	–	–	xx
Magnoliaceae	*Manglietia garrettii* Craib	x	–	x	x	xx
Melastomataceae	*Memecylon celastrinum* Kurz	x	–	x	x	–
Meliaceae	*Trichilla connaroides* (Wight & Arn.) Bentv. T	–	–	–	x	–
Moraceae	*Artocarpus gomezianus* Wall. ex Trec.	x	–	x	x	x
	*Artocarpus lakoocha* Roxb.	–	–	–	x	x
	*Ficus auriculata* Lour.	–	–	–	x	x
	*Ficus callosa* Willd.	–	–	–	x	x
	*Ficus religiosa* Linn.	–	x	–	–	x
Myrsinaceae	*Rapanea yunnanensis* Mez	x	–	x	x	xx
Myrtaceae	*Syzygium albiflorum* (Duthie & Kurz) Bahadur & R.C.Guar	x	x	–	x	xx
	*Tristaniopsis burmanica* (Griff) Peter G.Wilson & J.T. Waterh. var. rufescens (Hance) J.Parn. & Nic Lughadha	–	x	–	x	–
Pinaceae	*Pinus kesiya* Royle ex Gordon	x	–	–	–	x
	*Pinus merkusii* Royle. ex Gordon	x	x	–	–	x
Poaceae	*Bambusa membranacea* (Munro) C.M.A. Stapleton & N.H. Xia	–	x	–	–	x
	*Bambusa nutans* Wall.	–	x	–	–	x
	*Gigantochloa albociliata*	–	x	–	–	x
Proteaceae	Helicia nilagirica Bedd.	x	x	–	x	xx
Rubiaceae	*Canthium parvifolium* Roxb.	x	–	x	x	xx
	*Gardenia coronaria* Buch.-Ham	–	x	x	x	x
	*Gardenia obtusifolia* Roxb. Ex Kurz	–	x	x	x	x
	*Ixora cibdela* Craib	–	x	x	x	xx
	*Pavetta tomentosa* Roxb. ex. Sm. var. tomentosa	x	x	x	x	xx
	*Randia sootepensis* Craib	x	–	x	x	x
	*Tarrennoidea wallichii* (Hook.f.) Tirveng. & Sastre	x	–	x	x	–
	*Wendlandia tinctoria* A. DC.	x	x	x	x	x
Strychnaceae	*Strychnos nux-vomica* Craib	–	x	–	–	x
Styracaceae	*Styrax benzoides* Craib	x	–	x	x	xx
Theaceae	*Anneslea fragrans* Wall.	x	x	x	x	x
	*Camellia oleifera* Abel.	x	–	–	–	x
	*Camellia sinensis* Ktze. var. assamica Kitamura	–	–	–	x	x
	*Eurya acuminata* DC.	x	–	–	–	x
	*Eurya nitida* Korth.	x	x	x	x	x
	*Schima wallichii* Korth.	x	x	x	x	x
	*Ternstroemia gymnanthera* (Wight & Arn.) Bedd.	x	–	–	x	x

**Notes.**

x found xx found at genus level – not found

**Figure 4:**
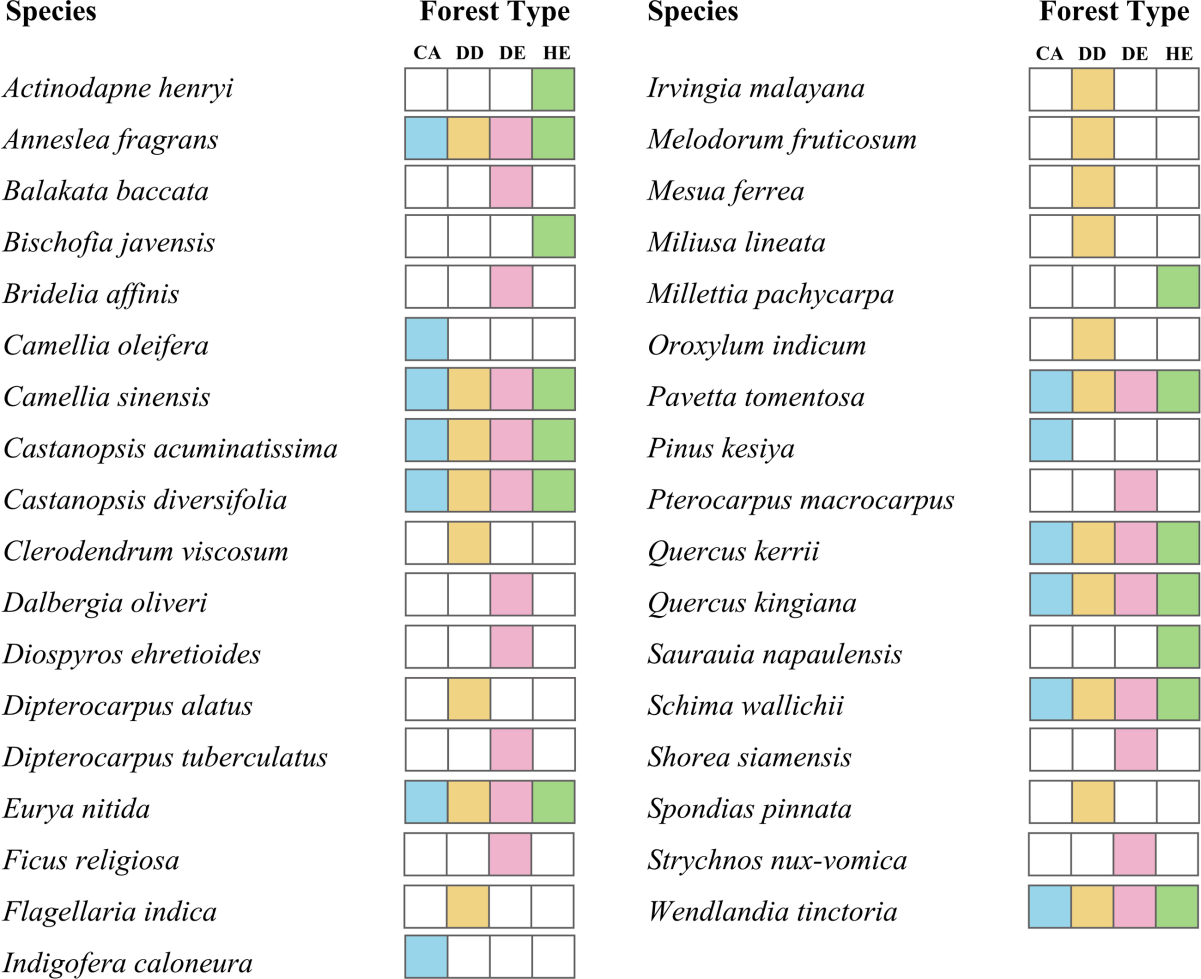
Plant species found in one type of the forest soil samples and species that detected in all four types of forest.

Furthermore, results from our study on the *trnL* region compared with the available sequences in GenBank showed that in a total of 1,225 plant species in Dipterocarpaceae and 1,101 species in Fagaceae family, there were only 268 (21.9%), and 154 (14.0%) species having *trnL* sequences deposited in the database, respectively, thus hindering the eDNA metabarcoding identification for these species. There are three, nine, eight and five plant species found only in the coniferous (CA), dry dipterocarp (DD), dry evergreen (DE) and hill evergreen (HE) community, respectively. Nine species were found in all forest types (Fig. 4). Interestingly, our molecular analyses showed traces of crops that were not expected to be found in the forest including lettuce (*Lactuca sativa*), maize (*Zea mays*), wheat (*Triticum timopheevii*) and soybean (*Glycine max*). Additionally, these crop plant species, which were found in the soil samples, were not visually detected *in situ* during the collection of the soil samples.

## Discussion

Herein, eDNA metabarcoding analysis revealed that unique plant groups are present in specific forest types, whereas some species were detected in all sample types. A strong congruence between the morphological- and eDNA-based data of plant diversity in the studied areas was also observed. The alpha diversity results indicate that Coniferous (CA) and Hill Evergreen (HE) samples present higher mean species diversity in comparison to Dry Dipterocarp (DD) and Dry Evergreen (DE) samples. Interestingly, morphological assessment only focusses on tree species and thus excludes the smaller plants like shrubs, herbs and grasses, such as *Alloteropsis*, *Canavalia, Germainia* and *Miscanthus*. However, the soil eDNA metabarcoding reveals both above- and below-ground species and assesses the composition and richness of plant communities from soil or sediment samples. Several studies reported higher eDNA-based diversity compared to traditional sampling approaches (Epp et al., 2012; Hiiesalu et al., 2012; Pansu et al., 2015; Alsos et al., 2018; Matesanz et al., 2019).

Two popularly used single-locus regions, *trnL* and *rbcL*, have been the markers of choice for plant eDNA metabarcoding. Although *trnL* gave better results of species richness in some studies, *rbcL* also showed higher sequence recovery of target taxa in others (Epp et al., 2012; Yoccoz et al., 2012; Fahner et al., 2016; Mallott, Garber & Malhi, 2018). Recently, the nuclear ribosomal internal transcribed spacer (ITS) is becoming popular marker of choice in the field, showing a high level of sequence divergence. The ITS2 region has greater coverage in vascular plant species sequences on GenBank when compared to the *rbcL* and *trnL* (Fahner et al., 2016). However, plant species have been misidentified as fungi in several studies given the limited nucleotide variation in the ITS2 region between fungi and plants. The main factor influencing eDNA metabarcoding results is the lack of relative DNA sequences in the databases (Edwards et al., 2018). For example, although *Castanopsis acuminatissima*, *Lithocarpus elegans*, and *Quercus kerrii* are common species in the study region, there was no available *trnL* reference sequence for these species, and thus could not be identified in our metabarcoding analysis. In addition, all 40 dominant plant taxa previously recorded in the Doi Suthep-Pui National Park lacked reference sequences in the GeneBank for *trnL* DNA barcoding region*,* thus none of them could have been identified through metabarcoding at species level in the soil samples (Supplementary data 1). DNA data of plants in Dipterocarpaceae and Fagaceae family are very scarce in the GeneBank. More comprehensive reference sequence database is required in order to increase species-level resolution for plant biodiversity assessments.

Herein, we report the identification of cultivated plant species, which were not visually detected during sampling. Plant DNA has been reported to remain in soil for up to 50 years and can be detected by metabarcoding (Yoccoz et al., 2012). The eDNA metabarcoding was able to detect crop plant species cultivated up to 8 years before soil sampling (Foucher et al., 2020). Interestingly, nearby lands and some areas in the Doi Suthep-Pui National Park used to be cultivated lands. Also, cultivated lands still exist within a 5–10 km radius of the forest areas where the soil cores samples were taken.

In addition, *Christisonia*, one of the rare plant genera of Thailand, was detected only in one replicate of soil samples collected from dry evergreen forest (DE). Inability to detect this species in some replicates might be due to the effects of PCR and variation in the soil material used for DNA extractions. Similar results have been previously published and thus sensitivity and reproducibility of the eDNA metabarcoding results have been a challenging issue (Boggs, Scheible & Meiklejohn, 2019). To avoid replicate variation, it has been suggested that performing technical replicates during PCR and pooling them before downstream processing could be effective (Lauber et al., 2013; Young, Weyrich & Cooper, 2016; Demanèche et al., 2017). *Christisonia* is a genus of root parasitic plants. The DNA concentration of these plants in the soil would be proportionally lower by the distance from host plant, as previous reported by (Osathanunkul, 2019) on an eDNA detection of a root parasitic species, *Sapria himalayana*. Some of the replicates of the DE sample were collected from areas far from the host plants, while the replicate in which *Christisonia* was detected was in close range. Therefore, the DNA concentration among the soil replicates was not equal and has led to variation in species detection.

The eDNA based method is more sensitive compared to the traditional morphology-based approaches. This is because it provides a comprehensive view of the targeted community, which is not only strongly reflecting current diversity, but also past biodiversity (Yoccoz et al., 2012; Smith et al., 2015; Foucher et al., 2020). Therefore, obtaining data from a soil DNA metabarcoding-based study would allow for better understanding of the studied areas. In our case, the presence of DNA from crop plant species in the soil samples collected from the forest still leads to a big question of whether we are now facing an agricultural intensification; however further investigation is needed.

## Conclusions

The eDNA metabarcoding is a rapid method for monitoring biodiversity, which could greatly advance assessment and enable understanding of the threats to the ecosystems, and could lead to effective conservation strategies. The method was proven to be revolutionary in biodiversity research. Herein, we report a strong congruence between the conventional morphology- and eDNA-based data of plant diversity in the studied areas. In addition, traces of crops that were not expected to be found in the forest were found in the eDNA analyses but were not identified by visual detection. eDNA metabarcoding used for biodiversity studies could allow the understanding of possible biodiversity shifts and give an insight towards land use change. Thus, the presence of crop plant traces probably suggests that we might face an agricultural intensification, which might lead to loss of biodiversity. Nevertheless, land reform for agricultural use should be well-planned in order to preserve forest biodiversity and in this context, the eDNA-based technology could be of paramount importance in assisting correct policy measures and planning.

## Supplemental Information

10.7717/peerj.11753/supp-1 Supplemental Information 1 Plant species found in traditional survey with no DNA barcode (*trnL* region)

## Additional Information and Declarations

### Field Study Permissions

The following information was supplied relating to field study approvals (i.e., approving body and any reference numbers): Field permits were granted by the Department of National Parks, Wildlife and Plant Conservation (field permit number: 0907.4/2529).

### Data Availability

The following information was supplied regarding data availability: The data is available at NCBI SRA: SRX10243284.
