# Supplementary material for: Exploring plant diversity through soil DNA in Thai national parks for influencing land reform and agriculture planning
Source: PeerJ. 2021 Aug 2;9:e11753. doi: 10.7717/peerj.11753 (PMC8340909; doi:10.7717/peerj.11753)
Supplement: Supplemental Information 1 [file peerj-09-11753-s001.docx]

**Supplementary data 1.** Plant species found in traditional survey with no DNA barcode (*trnL* region) deposited in GenBank

| **Family** | **Species** |
| --- | --- |
| Bignoniaceae | *Stereospermum neuranthum* Kurz |
| Burseraceae | *Protium serratum* Engler |
| Compositae | *Vernonia volkameriaefolia* Wall. ex DC. |
| Dipterocarpaceae | *Shorea obtusa* Wall. ex Blume |
| Ericaceae | *Vaccinium sprengelii* Sleumer |
| Euphorbiaceae | *Antidesma bunius* (L.) Spreng. var. bunius |
|  | *Antidesma sootepense* Craib |
|  | *Bischofia javensis* Blume |
| Fagaceae | *Castanopsis acuminatissima* Rehd. |
|  | *Castanopsis diversifolia* King |
|  | *Castanopsis purpurea* Barnett |
|  | *Lithocarpus elegans* Hatus. ex Soepadmo |
|  | *Lithocarpus finetii* A. Camus |
|  | *Lithocarpus lindleyanus* A. Camus |
|  | *Lithocarpus tenuinervis* A. Camus |
|  | *Lithocarpus thomsonii* Rehd. |
|  | *Quercus helferiana* A. DC. |
|  | *Quercus kerrii* Craib |
|  | *Quercus kingiana* Craib |
| Irvingiaceae | *Irvingia malayana* Oliv. ex A.W.Benn. |
| Lauraceae | *Actinodapne henryi* Gamble |
|  | *Cryptocarya pallens* Kosterm |
|  | *Lindera metcalfiana* Allen |

**Supplementary data 1. (Cont.)** Plant species found in traditional survey with no DNA barcode (*trnL* region) deposited in GenBank

| **Family** | **Species** |
| --- | --- |
| Leguminosae | *Acacia megaladena* Desv. var. megaladena |
|  | *Dalbergia ovata* Grah. |
|  | *Desnodium megaphullum* Zoll |
|  | *Indigofera caloneura* Kurz. |
|  | *Pterocarpus macrocarpus* Kurz |
| Magnoliaceae | *Manglietia garrettii* Craib |
| Melastomataceae | *Memecylon celastrinum* Kurz |
| Meliaceae | *Trichilla connaroides* (Wight & Arn.) Bentv. T |
| Myrsinaceae | *Rapanea yunnanensis* Mez |
| Myrtaceae | *Syzygium albiflorum* (Duthie & Kurz) Bahadur & R.C.Guar |
|  | *Tristaniopsis burmanica*(Griff) Peter G.Wilson & J.T. Waterh. var. rufescens (Hance) J.Parn. & Nic Lughadha |
| Proteaceae | Helicia nilagirica Bedd. |
| Rubiaceae | *Canthium parvifolium* Roxb. |
|  | *Ixora cibdela* Craib |
|  | *Pavetta tomentosa* Roxb. ex. Sm. var. tomentosa |
|  | *Tarrennoidea wallichii* (Hook.f.) Tirveng. & Sastre |
| Styracaceae | *Styrax benzoides* Craib |
